# Inducible expression of beta defensins by human respiratory epithelial cells exposed to *Aspergillus fumigatus *organisms

**DOI:** 10.1186/1471-2180-9-33

**Published:** 2009-02-11

**Authors:** Ludmila Alekseeva, Dominique Huet, Françoise Féménia, Isabelle Mouyna, Mahdia Abdelouahab, Adrien Cagna, Daniel Guerrier, Virginie Tichanné-Seltzer, Armelle Baeza-Squiban, René Chermette, Jean-Paul Latgé, Nadia Berkova

**Affiliations:** 1 UMR 956, INRA, AFSSA, ENVA, 22 rue Curie, 94700 Maisons Alfort Cedex, France; 2 Shemyakin and Ovchinnikov Institute of Bioorganic Chemistry, RAS; Moscow, Russian Federation; 3 Unité des Aspergillus, Institut Pasteur, 75724 Paris, France; 4 CNRS UMR 6061, Université de Rennes 1, 2 av. Pr. Léon Bernard, 35043 Rennes Cedex, France; 5 Laboratoire de Cytophysiologie et Toxicologie Cellulaire, Université Paris 7, 75251 Paris, France; 6 Service de Parasitologie-Mycologie, Ecole Nationale Vétérinaire d'Alfort, 7 av. de Général de Gaulle, 94704 Maisons Alfort Cedex, France

## Abstract

**Background:**

*Aspergillus fumigatus*, a saprophytic mould, is responsible for life-threatening, invasive pulmonary diseases in immunocompromised hosts. The role of the airway epithelium involves a complex interaction with the inhaled pathogen. Antimicrobial peptides with direct antifungal and chemotactic activities may boost antifungal immune response.

**Results:**

The inducible expression of defensins by human bronchial epithelial 16HBE cells and A549 pneumocyte cells exposed to *A. fumigatus *was investigated. Using RT-PCR and real time PCR, we showed an activation of hBD2 and hBD9 defensin genes: the expression was higher in cells exposed to swollen conidia (SC), compared to resting conidia (RC) or hyphal fragments (HF). The kinetics of defensin expression was different for each one, evoking a putative distinct function for each investigated defensin. The decrease of defensin expression in the presence of heat-inactivated serum indicated a possible link between defensins and the proteins of the host complement system. The presence of defensin peptide hBD2 was revealed using immunofluorescence that showed a punctual cytoplasmic and perinuclear staining. Quantification of the cells stained with anti hBD2 antibody demonstrated that SC induced a greater number of cells that synthesized hBD2, compared to RC or HF. Labelling of the cells with anti-hBD-2 antibody showed a positive immunofluorescence signal around RC or SC in contrast to HF. This suggests co-localisation of hBD2 and digested conidia. The HBD2 level was highest in the supernatants of cells exposed to SC, as was determined by sandwich ELISA. Experiments using neutralising anti-interleukine-1β antibody reflect the autocrine mechanism of defensin expression induced by SC. Investigation of defensin expression at transcriptional and post-transcriptional levels demonstrated the requirement of transcription as well as new protein synthesis during *A. fumigatus *defensin induction. Finally, induced defensin expression in primary culture of human respiratory cells exposed to *A. fumigatus *points to the biological significance of described phenomena.

**Conclusion:**

Our findings provide evidence that respiratory epithelium might play an important role in the immune response during *Aspergillus *infection. Understanding the mechanisms of regulation of defensin expression may thus lead to new approaches that could enhance expression of antimicrobial peptides for potential therapeutic use during aspergillosis treatment.

## Background

*Aspergillus fumigatus *(*A. fumigatus*) is a saprophytic mould that is responsible for life-threatening invasive pulmonary diseases in immunocompromised hosts. In general, *A. fumigatus *is propagated through airborne conidia [1]. Despite the availability of new antifungal drugs, the number of deaths due to invasive aspergillosis has progressively increased in the last decades with a rise in the number of immunosuppressed patients in modern clinical practices [2]. Therefore, a better understanding of the mechanisms responsible for resistance to *Aspergillus *infection is required. The respiratory epithelium plays an important role in the innate immune defence against various inhaled pathogens by sensing the signal from the external environment and stimulating the synthesis of the antimicrobial molecules directly affecting the microbes [3]. The defensin family of antimicrobial peptides is an evolutionary conserved group of small cationic peptides involved in the innate immune system of plants and animals. They are divided into α-, β- and θ-defensins, which differ from one another by the spacing and connectivity of their six cystein residues [4]. It was found that α-defensins are generally stored in the azurophilic granules of neutrophils and Peneth cells of the small intestine [5]. Defensins isolated from rhesus monkey neutrophils are referred to as θ-defensins because of their circular molecular structure [6]. Human β-defensins (hBD) are characteristic of epithelial tissue; they have been identified by traditional peptide purification, genomics-based searches [7-9] and an ORFeome-based peptide database search [10]. Some of these defensins are tissue-specific, whereas others are expressed in the epithelium of different origins: hBD1 is expressed in most epithelial cells [11,12], while hBD2 is most commonly expressed in the lung and thymus [13,14]. Newly discovered defensin hBD9 was found to be ubiquitously expressed in most tissues [10]. Inducible hBD2 expression by the epithelial cells exposed to microbial pathogens is well documented [15]. The direct killing of microorganisms has been ascribed to human defensins [7]. It was recently recognised that defensins have additional activities such as the chemoattraction of immature dendritic cells, T cells and monocytes, as well as activation of the professional antigen-presenting cells [16-18]. Killing of *A. fumigatus *by rabbit neutrophil cationic peptides [19], as well as antifungal activities of hBD2 against *A. fumigatus *[20], has been reported in *in vitro *experiments. Moreover, the expression of human drosomycin-like defensins, which display a broad spectrum of activity against *Aspergillus spp*, was found in several human tissues [21].

The role of the airway epithelium is not limited to the first mechanical barrier, but instead involves a complex interaction with *A. fumigatus *[22-24]. We hypothesized that various defensins may be expressed by the respiratory epithelium exposed to *A. fumigatus*. Taking the possibility into account that some host immunological reactions are *A. fumigatus *morphotype-specific [25,26], the present study has been designed to investigate the expression of defensins by human pneumocytes and bronchial epithelial cells exposed to different forms of *A. fumigatus*: RC, SC or HF. The expression of previously studied hBD1 [4] and hBD2 [14,15], as well as recently discovered hBD8, hBD9 and hBD18 [10], were analysed. Since hBD2 and hBD9 were found to be highly expressed by cells exposed to *A. fumigatus*, those defensins were chosen for further analysis in the current study. The inducible expression of hBD2 and hBD9 was revealed by RT-PCR in airway epithelial cells exposed to *A. fumigatus *organisms. Real time PCR demonstrated that the expression was higher in cells exposed to SC, compared to RC or HF. The presence of the intracellular hBD2 peptide was demonstrated using immunofluorescence. The HBD2 level was highest in the supernatants of cells exposed to SC, as determined by sandwich ELISA. Furthermore, it was found that transcriptional and post-transcriptional mechanisms are involved in the regulation of defensin expression. Detection of inducible defensin expression in human airway primary culture epithelial cells was proof of the biological significance of obtained results. Our finding that hBD2 and hBD9 are expressed and produced (hBD2) in human respiratory epithelial cells exposed to *A. fumigatus *is novel and indicates that respiratory epithelium might play an important role in the early immune response during *Aspergillus *infection.

## Results

### Expression of defensins by human pneumocytes and bronchial epithelial cells exposed to *A. fumigatus*

**Figure 1:**
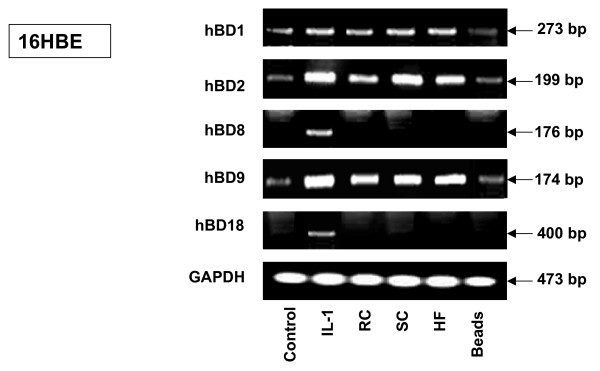
**RT-PCR analysis of various defensin expression levels in human 16HBE epithelial bronchial cells exposed to *A. fumigatus *organisms**. 16HBE human epithelial tracheal cells (5 × 10^6^) were grown in six well plates for 24 hours. After exposing the cells to RC, SC, HF or latex beads for 18 hours, the cells were washed with PBS, mRNA was isolated by TRIzol Reagent and RT-PCR was performed as described above in Materials and Methods. Specific primer pairs (Table 1) were used for RNA amplification: hBD1, 273 bp product; hBD2, 199 bp product; hBD8, 176 bp product; hBD9, 174 bp product; hBD18, 400 bp product and human GAPDH, which was used as an internal control, 473-bp product. All products were amplified according to the conditions described in Table 1. Cells were cultivated in a control well in the absence of *A. fumigatus*. As a positive control for defensin expression, exposure to human Il-1β was used in all experiments. The hBD1, hBD2 and hBD9 products were sequenced and confirmed to be identical to the predicted sequence. GAPDH was uniformly expressed. One of the four experiments is shown. Abbreviations: resting conidia (RC), swollen conidia (SC), hyphal fragments (HF), glyceraldehyde-3-phosphate dehydrogenase (GAPDH), interleukin-1β (Il–1β).

The expression of human defensins, hBD1 and hBD2, and newly described hBD8, hBD9 and hBD18, by the human pneumocytes A549 and bronchial epithelial cells 16HBE exposed to SC, RC or HF of *A. fumigatus *in the presence of Fetal Calf Serum was analysed by RT-PCR performed under the conditions presented in Table 1. The powerful defensin inductor, Il-1β, was used in experiments as a positive control. The cells were exposed either to 10^6 ^of *A. fumigatus *conidia, 20 μl of *A. fumigatus *HF solution, or 5 × 10^6 ^latex beads for 18 h. Compared to the control samples containing the untreated cells, an inducible expression of human beta defensins (hBD) 2, 8, 9 and 18 by 16HBE cells exposed to Il-1β was observed (Figure 1). Exposure of the cells to all of the morphotypes of *A. fumigatus *resulted in the strong inducible expression of hBD2 and hBD9, in contrast to the exposure of the cells to the 5 × 10^6 ^latex beads. The expression of hBD8 and hBD18 by cells exposed to *A. fumigatus *was not observed in the present study. The constitutive expression of human beta defensin1 (hBD1) was found in the current experiment. Since polymixin B drastically inhibits endotoxin activity, 20 μg of polymixin B per ml were added to cells before exposure to *A. fumigatus *organisms in some experiments, according to the method described by Mambula *et al*., in order to rule out endotoxin contamination [27]. This had no effect on defensin expression. Similar results were observed for A549 cells (data not shown). Therefore, hBD2 and hBD9 were chosen for further analysis of defensin expression by 16HBE and A549 cells exposed to *A. fumigatus*.

**Table 1:** Primer sequences, annealing temperatures and product size (RT-PCR).

Primers	Sequences	Conditions	Product size
hBD1f hBD1r	5'-agcgtctccccagttcctgaaatcct-3' 5'-tcttctggtcactcccagctcacttg-3'	38 cycles, 61°C	273 bp
hBD2f hBD2r	5'-catcagccatgagggtcttg-3' 3'-ggctttttgcagcattttgt-3'	38 cycles, 61°C, 2.5% DMSO	199 bp
hBD8f hBD8r	5'-tactcacctccagccttttgtcatcc-3' 5'-gggtgtagtgctctcaattcttggttg-3'	38 cycles, 61°C	176 bp
hBD9f hBD9r	5'-tgcagtaagaggtgatttgg-3' 5'-tgacatgataagtggtgttgg-3'	32 cycles, 56°C	174 bp
hBD18f hBD18r	5'-cctgcttcccaaggaccatgaaactc-3' 5'-ccgagaggaagtcatgagctatggtg-3'	38 cycles, 61°C	400 bp
GAPDHf GAPDHr	5'-cccatcaccatcttccagagc-3' 5'-ccagtgagcttcccgttcagc-3'	32 cycles, 61°C	473 bp

### Role of serum in defensin expression by human pneumocytes and tracheal epithelial cells exposed to *A. fumigatus*

**Figure 2:**
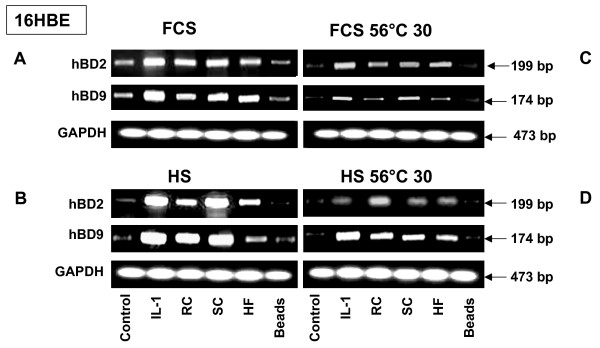
**RT-PCR analysis of defensin expression by 16HBE cells exposed to *A. fumigatus *organisms in the presence of different serums**. 16HBE human epithelial bronchial cells (5 × 10^6^) were grown in six well plates for 24 hours. The cells were then exposed to the different morphotypes of *A. fumigatus *or the latex beads in the presence of either Human (HS) or Fetal Calf Serum (FCS), (heated or not at 56°C). After 18 hours of incubation, the cells were washed with PBS, mRNA was isolated by TRIzol Reagent, and RT-PCR was performed as described above in Materials and Methods. Specific primer pairs (Table 1) were used for RNA amplification. The sizes of amplified products are indicated and were as predicted. All products were amplified according to the conditions described in Table 1. Cells were cultivated in a control well in the absence of *A. fumigatus*. GAPDH was uniformly expressed. One of the four results is shown.

In order to investigate the potential role of the serum and to set up the experimental conditions necessary for analysing the inducible expression of defensins by the human respiratory epithelium exposed to *A. fumigatus*, 16HBE and A549 human airway epithelial cells were incubated with *A. fumigatus *organisms (HF and SC or RC) or latex beads in the presence of either 10% heterologous Fetal Calf Serum (FCS) or 5% autologous human serum. Expression of hBD2 and hBD9 was evaluated. As a positive control, Il-1β was used in experiments. The cells were exposed to 10^6 ^of *A. fumigatus *conidia or 20 μl of *A. fumigatus *HF solution or 5 × 10^6 ^latex beads for various periods from 4 h to 18 h. The representative results of defensin expression by the 16HBE cells exposed for 18 hours to *A. fumigatus *are shown in Figure 2. Higher hBD2 and hBD9 gene expression was observed in the untreated control cells and the cells exposed to the latex beads in the presence of heterologous FCS (Figure 2A), compared to the intensity of bands corresponding to hBD2 and hBD9 in the cells incubated in the presence of 5% autologous human serum (Figure 2B). The treatment of the cells with Il-1β, as well as exposure of cells to either HF or conidia of *A. fumigatus*, strongly induced the expression of both defensins by the cells incubated with human serum (Figure 2B). Similar results were observed with A549 cells. The exposure of both types of cells to 10^5 ^conidia resulted in defensin expression as well (data not shown).

Taking the lower basal level of defensin expression into account in untreated control cells maintained in the medium containing human serum compared to FCS, all of the following experiments, unless otherwise specified, were performed with human respiratory cells incubated in the presence of 5% human serum.

The identities of hBD2 and hBD9 defensins were confirmed by direct sequencing of the products of predicted molecular weight generated by PCR amplification using upstream PCR primers.

### Effect of heat inactivation of serum on inducible defensin expression

The mechanisms of regulation of beta defensin expression by airway epithelial cells exposed to *A. fumigatus *organisms are unknown; the autocrine mechanism of defensin induction by cytokines cannot be ruled out. It was reported that *Aspergillus *induced cytokine production whereas heat inactivation of serum decreased cytokine production [28,29]. We therefore checked to see of the heat-labile serum factor was required for defensin expression. To do this, human 16HBE cells were incubated either with heterologous FCS or autologous human serum (previously heated or not at 56°C for 30 min) and simultaneously exposed for 18 hours either to *A. fumigatus *conidia, HF or the latex beads.

As shown in Figure 2, a high level of hBD2, as well as hBD9 expression, was observed in the cells incubated either with FCS (Figure 2A) or human serum (Figure 2B). Heat inactivation of any of the serums led to the partial decrease of the expression of both of the tested defensins by cells exposed either to *A. fumigatus *conidia, HF, or to Il-1β (Figure 2C, D).

### Kinetics of defensin expression by cells exposed to *A. fumigatus *organisms

**Figure 3:**
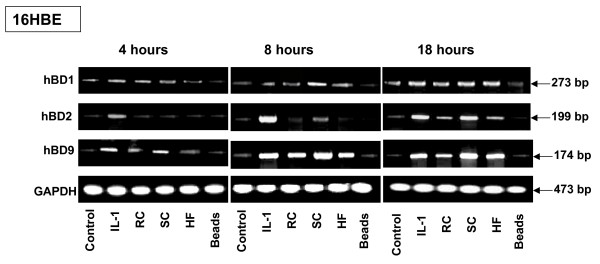
**Kinetics of defensin mRNA expression by 16HBE human epithelial bronchial cells exposed to *A. fumigatus *organisms**. 16HBE human epithelial tracheal cells (5 × 10^6^) were grown in six well plates for 24 hours. The cells were then exposed to the different morphotypes of *A. fumigatus *or latex beads for the different periods: 4 h, 8 h and 18 h. After incubation, the cells were washed with PBS, mRNA was isolated by TRIzol Reagent, and RT-PCR was performed as described above in Materials and Methods. Specific primer pairs and the conditions of RT-PCR are described in Table 1. The sizes of amplified products are indicated and were as predicted: hBD2, 199-bp product; hBD9, 174 bp product and human GAPDH, 473-bp product. The hBD2 and hBD9 products were sequenced and confirmed to be identical to the predicted sequence. Cells were cultivated in a control well in the absence of *A. fumigatus*. GAPDH was uniformly expressed. One of the four results is shown.

To analyse the kinetics of defensin expression, cells were exposed to *A. fumigatus *for 4, 8 and 18 hours, and the expression of hBD2 and hBD9 was examined. As a positive control, Il-1β-treated cells were examined. As a negative control, untreated cells or cells exposed to 5 × 10^6 ^latex beads were analysed as well. According to the results presented on Figure 3, the expression of both defensins, hBD2 and hBD9, were induced in the 16HBE cells treated with Il-1β either for 4, 8 or 18 hours. No hBD2 expression was detected after a 4-h exposure by 16HBE to SC, RC or HF of *A. fumigatus*, in contrast to hBD9 expression by cells exposed to all morphotypes of *A. fumigatus *for the same period. Incubation of the cells with both types of conidia or HF for 8 h resulted in a low level of hBD2 expression and a high level of hBD9 expression, comparable to expression by the cells treated with the positive control, Il-1β. Exposure of the cells to conidia or HF for 18 h led to the high expression of both defensins, hBD2 and hBD9. Exposure of the cells to the latex beads did not induce the defensin expression in any of the experiments. The constitutive expression of hBD1 by the cells exposed either to the different morphotypes of *A. fumigatus *or to the latex beads for the various periods was observed in the current experiment.

Similar kinetics of hBD2 and hBD9 expression was observed with A549 cells (data not shown).

### Real time PCR

**Figure 4:**
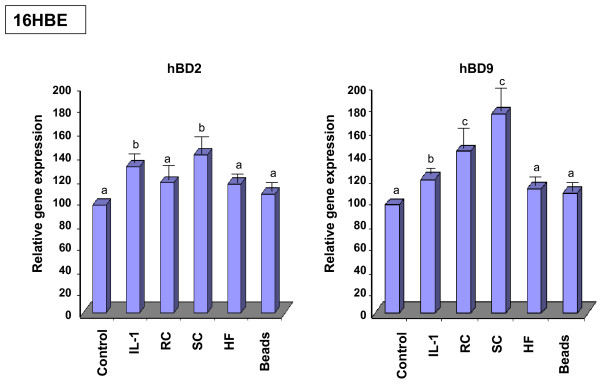
**Analysis of mRNA levels for HBD2 and HBD9 in 16HBE cells exposed to *A. fumigatus *organisms**. 16HBE cells (5 × 10^6^) were grown in six well plates for 24 hours. The cells were then exposed to the different morphotypes of *A. fumigatus *or latex beads for 18 h. Cells were cultivated in a control well in the absence of *A. fumigatus *or the latex beads. Isolation of total RNA and synthesis of cDNA was performed as described in Methods. Specific primer pairs and the conditions of real time PCR are described in Table 2. The level of mRNA for defensins was measured in total RNA preparation by quantitative real time PCR as described in Methods. Expression of all genes was normalised to the expression of the endogenous reference gene GAPDH. The expression value in control cells was used as the baseline. Data are calculated from three different experiments performed in triplicate. Means followed by the same letter are not significantly different.

The relative level of hBD2 and hBD9 expression in 16HBE and A549 cells exposed to different *A. fumigatus *morphotypes for 18 hours was quantified by real time PCR. The expression of both defensins was higher in Il-1β stimulated cells than in the control, as shown for 16HBE cells in Figure 4. Exposure of 16HBE cells to SC resulted in a statistically significant increase of hBD2 and hBD9 expression compared to that of the untreated control cells or the cells exposed to the latex beads. The increase of defensin expression was also found in the cells exposed to RC and HF. However, this difference was significant only for hBD9 in the cells exposed to RC. The difference in expression of hBD2 by the cells exposed to RC and in the expression of hBD2 as well as hBD9 by the cells exposed to HF did not reach a significant level. There was no difference between defensin expression in the untreated control cells and the cells exposed to the latex beads. Similar results were obtained with A549 cells.

### Neutralising anti-interleukine-1β antibody decreased defensin expression in cells exposed to swollen conidia

Since *A. fumigatus *has been shown to induce IL-1β in airway epithelium, and since the analysis of kinetic of defensin expression showed that the Il-1β-induced response was faster than the one induced by fungi (Figure 3), we investigated whether or not observed *A. fumigatus*-induced defensin expression was related to Il-1 β synthesized during anti-fungal response. For this reason, neutralising anti-interleukine-1β antibody was added to the cells before exposure to *A. fumigatus *organisms. One of the defensins, hBD-9, was chosen for real time PCR analysis of the role of Il-1 β in defensin expression. The results of real time PCR revealed that relative gene expression was statistically significantly decreased in the cells treated with anti-Il-1 β antibody before exposure to SC, compared to the cells only exposed to SC (120 ± 5 versus 143 ± 10 respectively). Relative gene expression was also decreased in the cells treated with anti-Il-1 β antibody before exposure to RC or HF, but the difference did not reach a statistically significant level. The pre-treatment of the cells with normal mouse immunoglobulin before exposure to *A. fumigatus *organisms had no effect on defensin expression.

### Analysis of defensin expression by human primary airway epithelial cells exposed to *A. fumigatus *conidia or hyphal fragments

**Figure 5:**
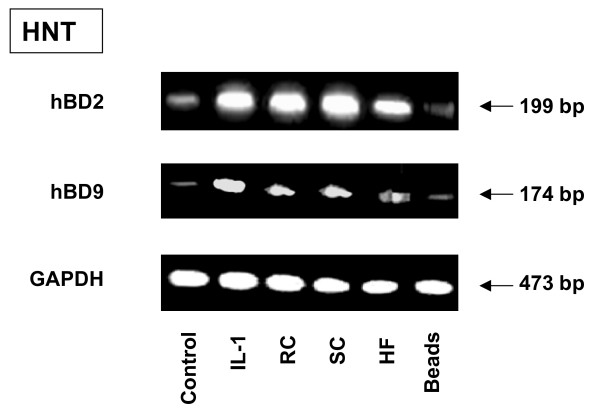
**RT-PCR analysis of defensin mRNA expression by primary epithelial cells**. Primary epithelial cells were obtained from human nasal turbinates (HNT), as described in Methods. The cells (5 × 10^6^) were grown in the six well plates for 48 hours. The cells were then exposed to either the latex beads or *A. fumigatus *organisms for 18 hours. The mRNA was then isolated by TRIzol Reagent and RT-PCR was performed as described above in Materials and Methods. Specific primer pairs (Table 1) were used for RNA amplification. The sizes of amplified products are indicated and were as predicted. The hBD2 and hBD9 products were sequenced and confirmed to be identical to the predicted sequence. GAPDH was uniformly expressed. Cells in a control well were cultivated in the absence of *A. fumigatus*. One of the three results is shown.

**Figure 6:**
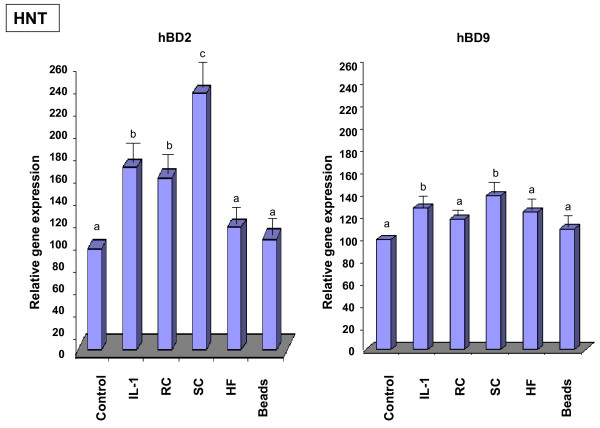
**Analysis of mRNA levels for HBD2 and HBD9 in HNT primary culture cells exposed to *A. fumigatus *organisms**. Primary epithelial HNT cells (5 × 10^6^) were grown in six well plates for 48 hours. The cells were then exposed to the different morphotypes of *A. fumigatus *or latex beads for 18 h. Cells were cultivated in a control well in the absence of *A. fumigatus *or the latex beads. Isolation of total RNA and synthesis of cDNA was performed as described in Methods. Specific primer pairs and the conditions of real time PCR are described in Table 2. The level of mRNA for defensins was measured in total RNA preparation by quantitative real time PCR as described in Methods. Expression of all genes was normalised to the expression of the endogenous reference gene GAPDH. The expression value in control cells was used as the baseline. Means followed by the same letter are not significantly different.

To provide evidence for the biological significance of the discovered phenomenon, we verified whether or not inducible defensin expression was observed in the human primary airway epithelial cells, in addition to the detected defensin expression in airway cell lines A549 and 16HBE (described above). Airway epithelial cells obtained from human nasal turbinates (HNT) of patients undergoing turbinectomy were exposed to RC, SC or HF or latex beads for 18 hours. Examination of hBD2 or hBD9 expression showed that both defensins were detected by RT-PCR in the primary culture cells exposed to all of the morphotypes of *A. fumigatus *(Figure 5). The relative level of hBD2 and hBD9 expression in HNT cells was quantified by real time PCR. The expression of both defensins was higher in Il-1β stimulated cells than in the control, as shown in Figure 6. Exposure of HNT cells to SC resulted in a statistically significant increase of hBD2 and hBD9 expression compared to that of the untreated control cells or the cells exposed to the latex beads. The increase of defensin expression was also found in the cells exposed to RC and HF. However, this difference was significant only for hBD2 in the cells exposed to RC. The difference in expression of hBD9 by the cells exposed to RC and in the expression of hBD2 as well as hBD9 by the cells exposed to HF did not reach a significant level. There was no difference between defensin expression in the untreated control cells and the cells exposed to the latex beads.

### Detection of the hBD2 peptide in human airway epithelial cells by immunofluorescence

**Figure 7:**
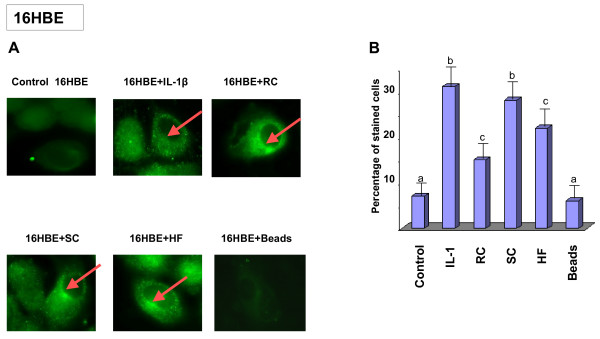
**Localisation of the hBD2 peptide in epithelial bronchial 16HBE cells**. 16HBE cells were seeded at 5 × 10^5 ^cells per well in 1 ml of DMEM/F12 on 18-mm-diameter cover slips in 12 well plates in triplicate and grown for 16 h at 37°C. After washing the cover slips with PBS-BSA, the cells were exposed to either latex beads, ethanol fixed conidia or ethanol fixed HF for 18 hours. Il-1β was used as a positive control. Following washing with PBS, the cells were fixed with a paraformaldehyde solution for 30 min at 37°C. The slides were then incubated in 1% BSA/PBS-Triton 0.05%, followed by a solution of 10% normal goat serum. After washing, polyclonal rabbit anti-human hBD2 at a dilution of 1:250 was applied as primary antibody overnight at 4°C, followed by incubation with FITC-labelled goat anti-rabbit secondary antibody at a dilution of 1:300 for 4 hours at room temperature. After washing, the cover slips were mounted on slides with ProLong antifade Vectashield. Samples were viewed with a Zeiss fluorescence microscope using ×400 magnification. The arrows indicate the cells stained with anti-hBD2 antibody. The percentage of stained cells was computed from triplicates of four experiments. Means followed by the same letter are not significantly different. +, presence; -, absence of Il-1β, *A. fumigatus *fixed organisms and latex beads. The punctuated localisation of the signal, which is concentrated adjacent to the nucleus (arrow), was observed. The data shown are representative of four independent experiments.

To determine if defensin peptides were present in the airway epithelial cells exposed to *A. fumigatus*, the hBD2 peptide was detected by immunofluorescence. Analysis of the hBD9 peptide was not performed since anti-hBD9 antibodies were not available. A549 or 16HBE cells were cultured on cover slips, subsequently exposed to either SC, RC, HF, latex beads or treated with Il-1β for 18 h, and stained with polyclonal anti-hBD2 antibody as described in Methods. As shown in Figure 7A, hBD2 was detected in the cytoplasm of airway epithelial 16HBE cells exposed to any of the morphotypes of *A. fumigatus*, but generally not in the untreated control culture or in the cells exposed to the latex beads, except for several individual cells that contained some amount of defensin peptides. These findings are consistent with the inducible expression of hBD2. Staining revealed the punctuated distribution of peptides in the cytoplasm with a concentration in the perinuclear region. It should be observed that the expression of the hBD2 peptide was not detected in each cell of the sample exposed to *A. fumigatus*. Quantification of the differences in the number of cells detected with anti-defensin-2 antibody showed that the number of stained cells in the untreated control culture was 8 ± 4%. The percentage of stained cells increased to 32 ± 4.6% after Il-1 β-treatment, to 17 ± 4.5% after exposure to RC, to 28 ± 5.2% after exposure to SC and to 20 ± 5.1% after exposure to HF, while exposure to the latex beads did not affect defensin expression (9 ± 3.9%) (Figure 7B). Similar results were obtained with A549 cells (data not shown).

### Co-localisation of hBD-2 and different *A. fumigatus *morphotypes

**Figure 8:**
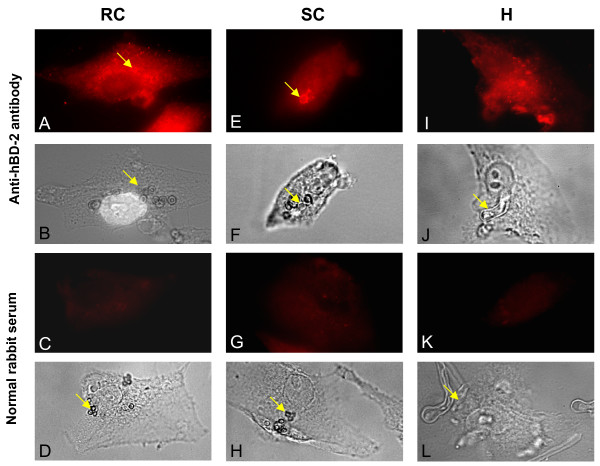
**Co-localisation of hBD2 and *A. fumigatus *organisms**. A549 cells were grown on cover slips for 16 h at 37°C. Cells were exposed to RC (A, B, C, D), SC (E, F, G, H) or HF (I, J, K, L) for 18 hours at 37°C. After fixation and permeabilisation, as described for Figure 7, cells were labelled with specific anti-hBD-2 antibody (A, B, E, F, I, J) and secondary antibody conjugated to Texas-red. Normal rabbit serum was used instead of anti-hBD2 as a negative control (C, D, G, H, K, L). Immunofluorescence signal (A, E, I, C, G, K) was compared to phase contrast image of the same cells (B, F, G, D, H, L). Arrows indicated different *A. fumigatus *morphotypes.

Previous experiments showed that human airway epithelial cells A549 internalised *A. fumigatus *conidia; a phagocytosis rate of 30% has been reported [30]. More then 50% of internalised conidia were found to co-localise after 24 hours with lysosomal proteins, CD63 and LAMP-1, which revealed the maturation of late endosome into lysosomes [31]. Similar results were obtained with primary human nasal epithelial cells. Staining of the cells with antibody against LAMP-1 demonstrated a positive immunofluorescence signal around digested *A. fumigatus *conidia [32]. Using the method described by these authors, we determined if different *A. fumigatus *morphotypes were co-localised with intracellular hBD-2. Labelling A549 cells with anti-hBD-2 antibody revealed cytoplasmic distribution of peptides. Comparison of the image of A549 cells stained by anti-hBD-2 antibody and the phase-contrast image revealed a positive immunofluorescence signal around resting (Figure 8A, B) or swollen (Figure 8E, F) conidia. This suggests a co-localisation of hBD2 and digested RC or SC. In contrast, no positive immunofluorescence signal was detected around HF, whereas the cells were positively stained with anti-human hBD2 antibody (Figure 8I, J). The normal rabbit serum control labels neither cytoplasm nor *A. fumigatus *morphotypes (Figure 8C, D, G, H, K, L). Similar results were obtained with 16 HBE cells.

### Quantification of hBD2 in cells supernatants by sandwich ELISA

**Figure 9:**
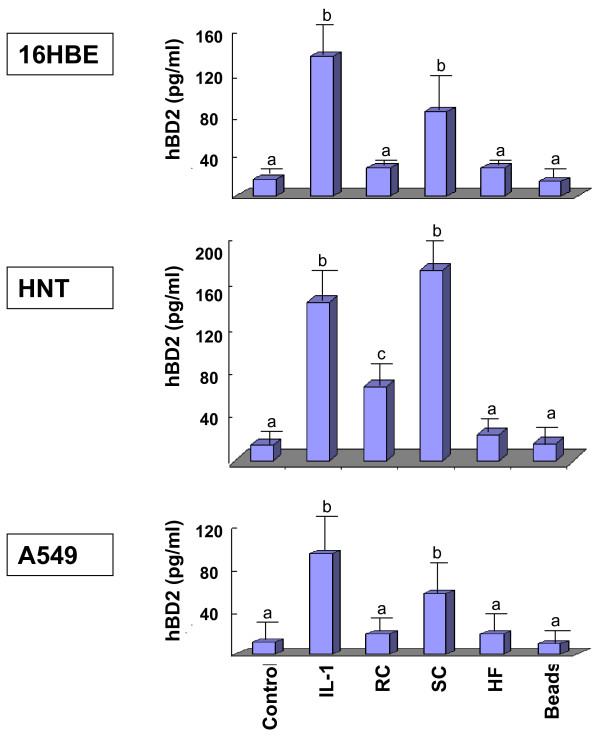
**Analysis of hBD2 level in cell supernatants**. The level of hBD2 in supernatants of 16HBE, A549 and primary culture HNT cells was measured by sandwich-ELISA. Briefly, cells were grown and exposed to different *A. fumigatus *organisms, latex beads or Il-1β (positive control) for 18 hours at 37°C. Supernatants were collected as described in Methods. The level of hBD was computed from duplicates of three experiments. Means followed by the same letter are not significantly different.

In order determine if synthesized hBD2 was released to cell supernatants, the level of hBD2 in the supernatants of 16HBE, A549 and HNT primary culture cells was evaluated by sandwich-ELISA. As shown in Figure 9, 140, 150 and 100 pg/ml of hBD2 were detected in the supernatants of Il-1β-treated 16HBE, HNT and A549 cells, respectively. Comparable levels of hBD2 were detected in the supernatants of all cells exposed to SC: 100, 180 and 70 pg/ml were found in the supernatants of 16HBE, HNT and A549 cells, respectively, which was statistically significantly higher then hBD2 levels in the supernatants of the cells alone or the cells exposed to RC, HF or latex beads. Exposure of any cells to RC or HF resulted in lower levels of hBD2, ranging from 20 to 70 pg/ml. The difference between hBD2 levels in the supernatants of the cells exposed to either RC, or those exposed to latex beads, was statistically significant for HNT cells, while this difference did not reach statistically a significant level for A549 and 16HBE cells. This could be explained by the different reactions of the different kinds of cells to the pathogen. The difference between hBD2 levels in the supernatants of 16HBE, HNT and A549 cells exposed to either RC, or those exposed to latex beads, was statistically insignificant.

### Analysis of hBD2 expression by airway epithelial cells exposed to live *A. fumigatus*

In order to determine if hBD2 expression was induced in the respiratory cells by live *A. fumigatus *organisms, RT-PCR and immunofluorescence analysis of cells exposed to unfixed 10^6 ^live conidia was performed. Using microscopic observation, we first examined the development of *A. fumigatus *in the environment of the epithelial A549 or 16HBE cells. When the RC were added to the epithelial cells, they settled onto the cells within 30 minutes and began to swell after 3–4 hours; after 8 hours of infection, the SC became polarised and began to germinate. The germ tubes then progressively elongated, forming the hyphae: after 18 hours of infection, the hyphae had completely covered the epithelial cells (data not shown).

**Figure 10:**
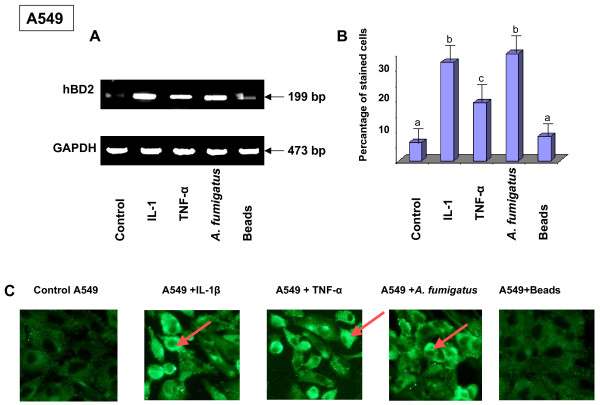
**Analysis of the defensin expression and its localisation in pneumocytes A549 exposed to live *A. fumigatus***. **A**. RT-PCR analysis of defensin mRNA expression by human pneumocyte A549 cells exposed to live *A. fumigatus*. A549 human epithelial bronchial cells (5 × 10^6^) were grown in six well plates for 24 hours. The cells were then exposed either to live *A. fumigatus *conidia or latex beads. After 18 hours of incubation, the cells were washed with PBS, mRNA was isolated by TRIzol Reagent, and RT-PCR was performed as described above in Methods. Specific primer pairs (Table 1) were used for RNA amplification. The size of the amplified product is indicated and was as predicted. Cells were cultivated in a control well in the absence of *A. fumigatus*. As an additional control, the cells were exposed to 10^6 ^latex beads for the same period. GAPDH was uniformly expressed. One of the four results is shown. **B**. Immunofluorescence detection of hBD2 in the A549 exposed to live *A. fumigatus *conidia. A549 cells were seeded at 5 × 10^5 ^cells per well in 1 ml of DMEM/F12 on 18-mm-diameter cover slips in 12 well plates in triplicate and grown for 16 h at 37°C. After washing the cover slips with 1%BSA/PBS, the cells were exposed to either latex beads or live *A. fumigatus *conidia for 18 hours. Il-1β was used as a positive control. Some cells were treated with TNF-α. Following washing with PBS, the cells were fixed with a paraformaldehyde solution for 30 min at 37°C. The slides were then incubated in 1% BSA/PBS, followed by a solution of 10% normal goat serum. After washing, polyclonal rabbit anti-human hBD2 at a dilution of 1:250 was applied as primary antibody overnight at 4°C, followed by incubation with FITC-labelled goat anti-rabbit secondary antibody at a dilution of 1:300 for 4 hours at room temperature. After washing, the cover slips were mounted on slides with ProLong antifade Vectashield. Samples were viewed with a Zeiss fluorescence microscope using ×400 magnification. The arrows indicate the cells stained with anti-hBD2 antibody. The percentage of stained cells was computed from triplicates of four experiments. Means followed by the same letter are not significantly different. +, presence; -, absence of Il-1β, TNF-α, live *A. fumigatus *organism and latex beads. The punctuated localisation of the signal, which is concentrated adjacent to the nucleus (arrow), was observed. The data shown are representative of four independent experiments.

RT-PCR analysis of the A549 cells exposed to live *A. fumigatus *RC for 4, 8 and 18 hours allows us to detect hBD2 expression after 18 hours of incubation (Figure 10A), whereas no inducible hBD2 expression was observed after 4 or 8 hours of incubation (data not shown). Treatment of A549 cells either with IL-1 β or TNF-α for 18 hours resulted in the inducible hBD2 expression. Detection of hBD2 in epithelial cells exposed to live *A. fumigatus *conidia for 18 hours by the immunofluorescence method revealed punctuated distribution of peptides in the cytoplasm with a concentration in the perinuclear region, similar to the staining of hBD2 in cells exposed to ethanol-fixed conidia or HF (Figure 10C). Several individual cells were stained with anti-hBD2 antibody in the untreated control cells or in cells exposed to the latex beads. Quantification of cells stained with hBD antibody revealed the increase in the number of stained cells from 6 ± 4.8% in the untreated control cells to 32 ± 5.7% in the IL-1β-treated culture, to 19 ± 6% in TNF-treated culture and to 35 ± 4.7% in the cells exposed to live *A. fumigatus*, compared to 8 ± 4% cells in the culture exposed to 5 × 10^6 ^latex beads (Figure 10B).

### Transcriptional and post-transcriptional mechanisms of defensin expression regulation

**Figure 11:**
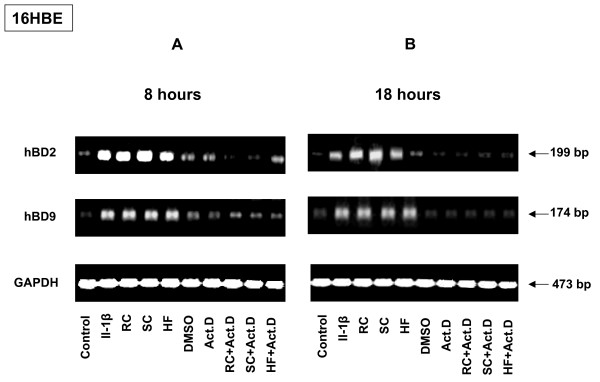
**Effect of RNA synthesis inhibition on inducible defensin expression**. 16HBE human epithelial bronchial cells (5 × 10^6^) were grown in six well plates for 24 hours. The cells were then pre-treated with 1 mg of actinomycin D/ml (ActD) or DMSO solvent for 1 h, and some samples were then exposed to the different morphotypes of *A. fumigatus *either for 6 (Figure 7A) or for 18 (Figure 7B) hours. There was no significant difference in viability between control and treated cells as assessed by staining with trypan blue. Furthermore, the yields of total RNA from the samples were compared and showed no difference. Total RNA was extracted and analysed by RT-PCR. The sizes of amplified products are indicated and were as predicted. GAPDH was uniformly expressed. Complete inhibition of hBD2 and hBD9 expression by the cells exposed to *A. fumigatus*, either for 6 or for 18 hours was observed after pre-treatment of the cells with actinomycin D.

In order to determine if the observed increase of defensin (hBD2 and hBD9) expression by cells exposed to *A. fumigatus *was related to transcriptional activation or enhanced stabilisation of mRNA, 16HBE cells were pre-treated with 0.5 μg of actinomycin D (an inhibitor of RNA transcription) per ml, or DMSO (vehicle control), 1 h before exposure of the cell to conidia or HF for an additional 8 or18 h, as described in the literature [33]. The viability of 16HBE cells and total RNA yield were verified after each treatment, and there was no difference between treated and untreated control cells. As shown in Figure 11, exposure of the 16HBE cells either to DMSO or Act D resulted in almost no increase of defensin expression compared to control cells, while the expression of both defensins by the 16HBE cells exposed to the various forms of *A. fumigatus *conidia for either 8 or 18 h was inhibited by the pre-treatment of cells with Act D. Therefore, the data indicated that new gene transcription is required for hBD2 and hBD9 expression by cells exposed to *A. fumigatus *RC, SC or HF.

**Figure 12:**
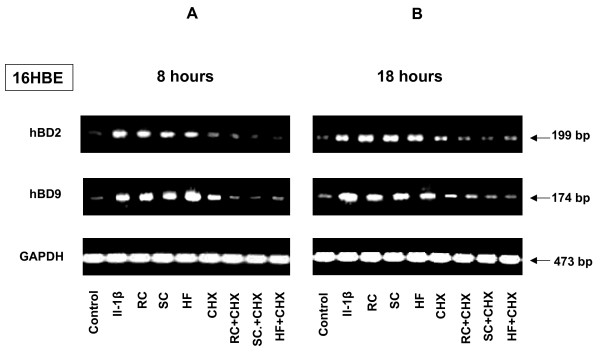
**Effect of protein synthesis inhibition on inducible defensin expression**. 16HBE human epithelial tracheal cells (5 × 10^6^) were grown in six well plates for 24 hours. The cells were then pre-treated with 2.5 μg of cycloheximide (CHX) for 1 h, and some samples were then exposed to the different morphotypes of *A. fumigatus*, either for 6 (Figure 8A) or for 18 (Figure 8B) hours. There was no significant difference in viability between control and treated cells as assessed by staining with trypan blue. Furthermore, the yields of total RNA from the samples were compared and showed no difference. Total RNA was extracted and analysed by RT-PCR. The sizes of amplified products are indicated and were as predicted. GAPDH was uniformly expressed. Complete inhibition of hBD2 and hBD9 expression by the cells exposed to *A. fumigatus *either for 6 or for 18 hours was observed after pre-treatment of the cells with cycloheximide.

To determine if the increase in defensin mRNA expression was dependent on protein synthesis, 16HBE cells were pre-treated with 2.5 μg of cycloheximide (CHX), a protein synthesis inhibitor, 1 h before exposure to *A. fumigatus*. Pre-treatment of the cells with only CXH did not change defensin expression, compared to control cells. In contrast, pre-treatment of 16HBE cells with CXH resulted in the inhibition of defensin expression after exposure to *A. fumigatus *(Figure 12). Therefore, it could be hypothesized that protein synthesis might be required for induced accumulation of defensin mRNA.

## Discussion

A better understanding of the mechanisms responsible for the defence against invasive *Aspergillus *infection is required to develop strategies aimed at boosting the antifungal actions of the immune system. Defensins, or antimicrobial peptides, which are implicated in potentiating innate and adaptive immunity [16-18] in addition to direct antimicrobial activities [20], would be a good candidate as a therapeutic agent for enhancing host defence mechanisms. Since the invasion of the airway epithelium by *A. fumigatus *conidia may play an important role in the development of aspergillosis, we therefore investigated the involvement of defensins in the response of pneumocytes A549 and bronchial epithelial cells 16HBE exposed to *A. fumigatus *in this study. The expression of human defensins hBD1, hBD2, hBD8, hBD9 and hBD18 was analysed. In agreement with earlier findings [34], constitutive expression of hBD1 by the epithelial cells 16HBE and A549 was observed in our experiments. It was found that hBD2 and hBD9 are highly expressed by the epithelial respiratory cells exposed to SC, RC or HF of *A. fumigatus*, while hBD8 and hBD18 gene expression was not observed in the current study. Previous investigations revealed that hBD2 was induced by various stimuli including microbes, cytokines and growth factors [33,35]. Inducible expression of hBD2 defensins by airway epithelial cells exposed to *A. fumigatus*, observed in the present work, is therefore in agreement with earlier observations. The role of the recently discovered hBD9 in innate antimicrobial defence is not well determined; however, hBD9 gene regulation in gingival keratinocytes exposed to *Candida albicans *has been described [36]. Additional investigations are essential for a better understanding of its role in direct antimicrobial activity and its contribution to innate immunity. The role of hBD8 and hBD18 in innate immunity of respiratory epithelium exposed to *A. fumigatus *cannot be ruled out before evaluation of other epithelial respiratory cells or other induction conditions. Further analysis of those defensins is recommended.

We have shown that the maintenance of the human epithelial cells in the presence of preheated serum (56°C for 30 min) resulted in the partial decrease of defensin expression. Involvement of the heat-labile serum factor suggests the potential role of the complement for defensin expression. The possible link between the proteins of the complement system and defensin expression may be anticipated since the interaction between the defensins and proteins of the complement system was demonstrated. It was found that HBD2 inhibits the classical pathway of the complement system [37]. Moreover, the interrelationship between the respiratory tract and the complement system was studied in an animal model [38]. The origin of complement proteins present in the lining fluid of the respiratory tract is thought to result mainly from plasma that exudes into the bronchoalveolar space. However, it was shown that human bronchial epithelial cells generate complement protein C3: the modulation of its expression by proinflammatory cytokines might be an additional regulatory mechanism of local airway defence during infection [39].

Furthermore, the kinetics of the expression of human beta defensins, hBD2 and hBD9, by airway epithelial cells exposed to the deferent morphotypes of *A. fumigatus *was analysed. Analysis of the kinetics of hBD2 and hBD9 defensin expression by cells exposed to *A. fumigatus *showed the prompt inducible expression of hBD9, following by delayed hBD2 expression. This could allow us to hypothesize that the host immune system may react against *A. fumigatus *by using the cascade of newly synthesized defensins that probably possess the different functions. However, this hypothesis would require further investigation at the protein level. Our data are in agreement with the analysis of kinetics of hBD2 expression by A549 cells exposed to *Mycobacterium tuberculosis*; infection of A549 cells resulted in hBD2 gene expression as early as 6 hours postinfection, while maximum expression was detected at 18 and 24 hours postinfection [35].

Several lines of evidence eliminated the possibility that observed inducible defensin expression was related to endotoxin contamination of *A. fumigatus *organisms. First, the addition of Polymixin B (known for its endotoxin-neutralising activity) to the cells prior to exposure to *A. fumigatus *organisms did not have any effect on defensin expression. Second, rigorous measures were undertaken to keep endotoxin out of the experimental system, including washing of *A. fumigatus *organisms with the solution containing Polymixin B during preparation, utilisation of endotoxin-free plasticware and solutions in experiments, and washing of fungal organisms in endotoxin-free PBS prior to use.

The expression of hBD2 and hBD9 was found to be higher in A549, 16HBE and primary culture HNT cells exposed to SC compared to RC or HF, as shown by quantitative PCR. During asexual growth, the morphological form of *A. fumigatus *changes from resting to swollen conidia, which then form germ tubes that continue growing in hyphal form. These transformations are accompanied by the modification of surface structures. The RC are covered with a proteinoceous layer that is disrupted during swelling, exposing the inner layer. Therefore, the surfaces of various *A. fumigatus *morphotypes differ form each other and, consequently, the reaction of host cells may vary towards divergent *A. fumigatus *growth forms [40]. Our findings suggest that infected hosts can discriminate between inactive RC and active potentially-invasive SC. The data are consistent with findings showing that SC (the mature form of *A. fumigatus*), but not RC-activated NF-kβ, stimulated pro-inflammatory cytokines and the production of reactive oxygen by host macrophages [40].

Moreover, the presence of the hBD2 peptide in the respiratory cells was investigated. Detection of the hBD2 peptide by immunofluorescence in A549 and 16HBE cells exposed to the different forms of *A. fumigatus *confirmed its inducible expression in the infected cells. The presence of the negatively-stained cells in the infected culture may be due to defensin synthesis in the subpopulation of the epithelial cells or because of the release of synthesized defensins by the activated cells. The detection of the beta-defensin hBD2 peptide in the individual unstimulated control cells is in agreement with the observation made for the alpha-defensins; it has been reported that individual untreated HL-60 cells may contain variable amounts of alpha defensin, as assessed by immunostaining [41]. Undoubtedly, inducible expression of defensin by cells exposed to *A. fumigatus *may represent the recruitment of additional cells that would synthesize antimicrobial peptides and further upregulation of defensin synthesis in cells that originally contained defensin. Punctuated distribution of peptide in the cytoplasm of A549 and 16HBE cells with a concentration in the perinuclear region was similar to the staining of defensin expressed by human gingival epithelial cells exposed to cell wall extract of the gram-negative periodontal bacteria, *Fusobacterium nucleatum *[33], suggesting that the mechanism of defensin expression may be universal for the different infectious agents. The punctuated perinuclear pattern of immunostaining may be related to the localisation of hBD2 in the endoplasmic reticulum and Golgi apparatus, which is in agreement with the previous observations of Rahman et al., showing that the hBD2 peptide was expressed in rough endoplasmic reticulum, the Golgi complex and cytoplasmic vesicles of human colon plasma cells [42]. Quantification of the cells stained with anti-hBD2 antibody revealed that SC induced a greater number of cells that synthesized hBD2, compared to RC and HF. Analysis of hBD2 levels in the supernatants of A549, 16 HBE and primary culture HNT cells confirmed this observation; significantly higher hBD2 levels were detected in all tested cell supernatants exposed to SC, compared to those exposed to RC, HF or latex beads. The level of hBD2 detected in the cell supernatants does not exceed 200 pg/ml and was comparable to the level of hBD2 in cell supernatants exposed to other pathogens [43]. Since Okamoto et al. showed that the effective dose of synthetic hBD2 was 1.5 μg/ml, we can hypothesize that the chemotactic activity of hBD2 rather then its direct antifungal activity plays a more important role in the protection of the infected host [20]. However, antifungal activity of defensins in synergy with other antifungal factors *in vivo *cannot be excluded.

Co-localisation analysis of hBD2 and *A. fumigatus *morphotypes allow us to detect RC or SC stained with hBD2 antibody in contrast to HF; these observations confirm the different mechanism of hBD2 induction by various morphotypes. Our findings are in agreement with the observations of Lopez Bezzera et al. who found that *A. fumigatus *conidia and hyphae injure endothelial cells via different mechanisms [44]. This difference between the different growth phases of *A. fumigatus *could be due to the discrepancy of the mechanisms of defensin induction, which may possibly be related to the diverse types/numbers of molecules involved in this process. Immunofluorescence analysis of inducible hBD2 expression by cells exposed to live *A. fumigatus *organisms revealed the perinuclear staining of peptide, similar to the staining observed in cells exposed to fixed *A. fumigatus*, pointing to the biological significance of our findings. Given the fact that conidia germinate and form hyphae after epithelial cells are exposed to live *A. fumigatus *conidia for 18 hours, in agreement with previous observation [44], we can then hypothesize that defensin expression is possibly induced by different morphotypes in this experiment.

Our observations of the induced defensin expression in the airway epithelial cells treated with Il-1 β or TNF-α, the cytokines that play an important role during *Aspergillus *infection [45,46], suggest that defensin expression in infected cells may be induced by *A. fumigatus *organisms, as well as by the cytokines involved in the infectious process. Therefore, the regulation of defensin expression during *Aspergillus *infection may possibly depend on a variety of factors.

Significant decrease of defensin expression by neutralising anti-IL-1β antibody, added to the cells prior exposure to SC, reflects the autocrine mechanism of defensin induction. A statistically insignificant decrease of defensin expression in the cells treated with anti-IL-1β antibody and exposed to RC or HF supported the hypothesis that the host immune system may distinguish and react differently towards divers *Aspergillus *morphotypes.

Finally, to better understand defensin synthesis, we investigated the involvement of transcriptional and post-transcriptional mechanisms in the regulation of defensin synthesis. The inducible expression of hBD2 and hBD9 by cells exposed to all morphotypes of *A. fumigatus *was inhibited by pre-treatment with actinomycin D, implying that defensin genes are regulated at the transcriptional level. The inhibitory effect of cycloheximide on defensin induction substantiates the requirement for new protein synthesis, which may include cell receptors, transcription factors or proteins that enhance mRNA stability. Thus, post-transcriptional mechanisms of regulation were involved in the inducible expression of defensins as well.

## Conclusion

While the direct fungicidal activity of hBD2 against *A. fumigatus *was revealed in the *in vitro *model [20], this is the first study, according to our knowledge, showing hBD2 and hBD9 defensin expression by host airway epithelial cells exposed to *A. fumigatus*. Defensin expression was higher in the cells exposed to SC than to RC or HF. Moreover, the HBD2 level was elevated in the supernatants of cells exposed to SC, compared to other *Aspergillus *morphotypes. Our findings suggest that identification of the most invasive fungal form by the host may be beneficial for anti-fungal host response.

Autocrine regulation of defensin expression in cells exposed to *A. fumigatus *was established in the experiments with neutralising anti-Il-1β antibody. Investigation of defensin expression at transcriptional and post-transcriptional level demonstrated the requirement of transcription as well as new protein synthesis during *A. fumigatus *defensin induction. The presence of defensin peptide hBD2 was revealed using immunofluorescence that showed a punctual cytoplasmic and perinuclear staining, suggestive of endoplasmic reticulum and Golgi apparatus localisation. The discovery of inducible hBD2 and hBD9 defensin expression by human primary respiratory culture cells is indicative of the biological significance of the observation. Our finding provides evidence that respiratory epithelium might play an important role in the early immune response during *Aspergillus *infection. Taking the antimicrobial activity of defensins together with their capacity to induce the migration of cells involved in the immune response into account, we can hypothesize that defensins may link innate and acquired immunities of the host infected by *A. fumigatus*. Future study of the regulation of defensin expression might provide new approaches that may enhance expression of antimicrobial peptides for potential therapeutic use during aspergillosis treatment.

## Methods

### Reagents

Human serum, actinomycin D and cycloheximide were obtained from Sigma. Actinomycin D and cycloheximide were dissolved in dimethyl sulfoxide (DMSO) (Sigma). In all the experiments, the concentration of DMSO was always less than 0.1% (vol/vol). Interleukun-1β (Il-1 β) was purchased from Sigma. Lyophilised powder of Il-1β was reconstituted to the stock concentration of 10 μg/ml with sterile phosphate buffered saline (GIBCO BRL). Twenty ng/ml of IL-1β solution was used as a positive control for defensin expression in all experiments. Monoclonal anti human Il-1 β antibody (I3642) were obtained from Sigma. Recombinant human Tumor Necrosis Factor alpha (TNF-α) was obtained from Sigma; 10 ng/ml of TNF solution was used in the experiments.

Anti-hBD-2 polyclonal antibody was purchased from Peptide International, Inc (Louisville, Kentucky, USA). Lyophilised powder of anti-hBD-2 antibody was reconstituted to the stock concentration of 10 mg/ml with sterile phosphate buffered saline (GIBCO BRL). Bronchial epithelium medium (BEGM) was obtained from Lonza Group Ltd (Basel, Switzerland).

### Maintenance of endotoxin-free conditions

Experiments were designed to minimise endotoxin contamination by using purchased endotoxin-free plasticware and heating all glassware at 180°C for 4 hours. All solutions used in the experiments contained less then 0.007 endotoxin unit/ml (minimal detectable level) when tested with Limulus amebocyte lysate assay (Sigma). *A. fumigatus *organisms were washed in the solution containing Polymixin B during preparation.

### Patient material

Human nasal turbinates of patients undergoing turbinectomy (Pr. G. Lamas, La Pitié-Salpêtrière University Hospital Centre, Paris, France) were used for the preparation of the primary epithelial cells. All patients signed an informed consent form before participating in this research protocol, which was approved by the Institutional Ethics Committee.

### Fungal strain and growth conditions

The A. *fumigatus *strain, CBS 144.89 (Institut Pasteur, Paris, France), was used throughout this study. *A. fumigatus *conidia were prepared as previously described [22]. Briefly, conidia of *A. fumigatus *were obtained from cultures grown on YM agar (0.3% yeast extract, 2% malt extract, 0.5% peptone and 0.5% agar) for three days at 37°C. Conidia were harvested by flooding the plates with sterile distilled water and then suspending the hydrophobic conidia in 0.01% Tween 20 in phosphate-buffered solution (PBS). To remove hyphae and debris, the conidial suspension was filtered through four levels of gauze. The RC obtained were maintained at 4°C.

### Preparation of swollen conidia and hyphal fragments

SC were prepared as described [47]. Briefly, 5 × 10^9 ^of resting *A. fumigatus *conidia were incubated in 200 ml of Sabouraud medium for 5 hours at 37°C in order to obtain the isodiametric swelling of the conidium resulting in the development of SC. As demonstrated by microscopic examination, the majority of the organisms were single conidia, with a few small clumps containing two to four organisms. To obtain a homogeneous preparation, the suspension was gently sonicated for 10 seconds using a Branson Sonifier 450 (output level 2; Branson Ultrasonics, Danbury, CT, USA). Before exposure of the cells to conidia, the solution was vigorously vortexed and observed microscopically to ensure the absence of clumps.

Hyphal fragments (HF) were prepared by incubating 2 × 10^8 ^of resting conidium in 200 ml of Sabouraud medium for 18 hours at 37°C with shaking in order to obtain a homogenous solution of the small HF. The tubes were then centrifuged in order to spin down the pellet. For the purpose of standardising the hyphal inoculum by weight, the pellet, which almost exclusively contained mycelium, was washed twice with Hanks balanced saline solution without Ca2+ and Mg2+ (HBSS w/o), followed by filtration through the gauze that was weighed beforehand. The gauze containing HF was dehydrated at 60°C overnight and weighed [29]. The difference between the weight of the gauze alone and the gauze containing the dry mycelium corresponds to the weight of the dry mycelium. 700 mg of dry weight of mycelial mass was obtained during experiments under the conditions described above. Twenty ml of PBS were then added to the dry mycelial mass and vigorously resuspended. All *A. fumigatus *morphotypes were prepared so as to minimise endotoxin contamination as described [27]. To eliminate potential endotoxin contamination, RC, SC or HF were washed in PBS containing 50 μg/ml of Polymixin B, known for its capacity to drastically decrease endotoxin activity, followed by four additional washings in endotoxin-free PBS. Since human cells have to be exposed to the different forms of *A. fumigatus *for various periods of time (including 18 hours to allow the RC to germinate), all *A. fumigatus *morphotypes were fixed in ethanol. The different solutions, containing RC, SC or HF, were centrifuged and resuspended in a 70% solution of ethanol in PBS and stored in a refrigerator for 24 hours as described in the literature [29]. After centrifugation, either conidium or HF were vigorously resuspended in PBS containing 10 mg of RNAse A per ml (Sigma Aldrich) and incubated for 30 min at 37°C to remove intracellular RNA [29]. After several washings in PBS, the different forms of *A. fumigatus *were viewed under the microscope; homogeneous solutions containing single resting or SC were obtained. The morphology of the mycelium was not altered. After being fixed in ethanol, mycelia (700 mg of dry weight in 20 ml of PBS) were used as a standard HF solution. In experiments with ethanol-fixed *A. fumigatus *organisms, the equivalent volume of the supernatant from the last washing was added to the human cells to check for the release of any toxic material as a result of the ethanol treatment. There was no induction of the defensin expression in the cell culture incubated in the presence of the supernatants from the last washing.

### Human cell lines and growth conditions

A type II pneumocyte cell line A549 derived from a human lung carcinoma was obtained from the American Type Culture Collection [ATCC CCL 185 [48]] and maintained in Kaighn's modification of HAM's F12 medium supplemented with 10% FCS (Invitrogen, Cergy Pontoise, France), pen/strep (16 mg/ml penicillin and 100 mg/l streptomycin), 2 mM L-glutamine and 1.5 g/l sodium bicarbonate. The cells were grown until confluent at 37°C in an incubator with a humidified atmosphere of 5% CO2. Trypsin/EDTA (Invitrogen) was used to release adherent cells for subculturing when this was required.

Human bronchial epithelial SV40-transformed cells (16HBE) were kindly provided by Dr. D.C. Gruenert (Research Facility, California Pacific Medical Center, San Francisco, CA, USA). 16HBE cells were maintained in DMEM/F12 medium (Invitrogen) with 10% FCS (Invitrogen), pen 100 U/ml/strep 100 μg/ml, 2 mM L-glutamine (Sigma) and 1 Ug/ml de fungizone and 1.5 g/l sodium bicarbonate (Sigma), and were grown until confluent [49].

### Establishment and maintenance of human airway epithelial primary culture cells

Primary epithelial cells were obtained from human nasal turbinates (HNT) of patients undergoing turbinectomy as previously described [50]. Briefly, HNT were washed in Dulbecco's modified Eagle medium DMEM/F12 (Invitrogen) and incubated with 2 mg/ml pronase (Protease XIV; Sigma,) in DMEM/F12 supplemented with pen/strep, at 4°C for 16–20 h under slow rotary agitation (80 rpm.). After washing, aggregates were discarded and dissociated cells were filtered using a 30-μm pore filter. The cell suspension was then plated for 2 h at 37°C on plastic dishes (Falcon) to eliminate contaminating fibroblasts. After centrifugation, the supernatant containing the epithelial cells was cultivated in a 1:1 mix (vol:vol) of bronchial epithelium medium BEGM (Lonza Ltd): DMEM/F12 supplemented with Clonetics singlequots (5 μg/mL insulin, 0.5 μg/mL hydrocortisone, 0.5 μg/mL epinephrine, 6.5 ng/mL triiodothyronine, 10 μg/mL transferrin, 0.5 ng/mL human epidermal growth factor, 50 μg/mL gentamicin-amphotericinB, 0.13 mg/mL bovine pituitary extract), 50 U/mL of penicillin-streptomycin and 0.5% fungizone.

### Heat inactivation of the serum

In the experiments devoted to the investigation of the role of the heat-labile component of serum in the production of defensins by the human airway epithelium, heat inactivation of the serum, the recognised method for serum decomplementation, was performed as described [51]. Briefly, either human autologous serum or heterologous FCS was heated at 56°C for 30 min. After cultivation of the human respiratory cells under the conditions described above, the cells were exposed to *A. fumigatus *in the medium containing serum that was either heat-inactivated or not.

### Exposure of the cells to *A. fumigatus *conidia or hyphal fragments

5 × 10^6 ^of A549, 16HBE or primary culture cells were placed in six well plates in 1.5 ml of the corresponding medium described above and grown until confluence. Following washing of A549, 16HBE or primary culture cells with PBS, 10^6 ^of *A. fumigatus *conidia per millilitre of medium were added to the cells for 4, 8 or 18 hours. Exposure to HF was carried out by incubation of the cells for 4, 8 or 18 hours with 20 μl of the standard solution (35 mg of dry weight/ml) obtained from 2 × 10^8 ^of resting conidium as described above. All *A. fumigatus *morphotypes were washed an additional four times in endotoxin-free PBS prior to use to eliminate potential endotoxin contamination.

After incubation, unbound conidia were removed by washing wells with PBS prior to RNA purification.

Primary epithelial cells were seeded at 5 × 10^6 ^cells per well and grown for 48 h before exposure to *A. fumigatus*.

In some experiments, the cells were exposed to 10^6 ^unfixed live conidia for 18 hours.

To be sure that the inducible expression of defensins was specific to *A. fumigatus *and did not simply reflect a phagocytosis response, latex beads were used as a control, since it was shown that the respiratory cells are capable of internalising nonspecific particles such as latex beads [52]. Compared to the concentration of conidia, up to a five-fold higher concentration of latex beads was used in the experiments, as suggested [30].

Before exposing the cells to the *A. fumigatus *organisms, the solutions were vigorously vortexed and observed microscopically to ensure that they did not contain clumps.

### RNA isolation and analysis of defensin expression by RT-PCR

In order to ensure that the cells were exposed to different morphotypes of *A. fumigatus *organisms (conidia or HF) during the incubation period, the cell culture was observed microscopically at the beginning and at the end of the exposure. The medium was discarded, the wells were briefly washed with PBS solution, and TRIzol reagent was added to the cells.

Total RNA was isolated with TRIzol Reagent (Invitrogen, Cat N 15596-026) according to the manufacturer's instructions. RNA was precipitated with ethanol and resuspended in diethyl pyrocarbonate H_2_0. The RNA concentration was measured by spectroscopy, and the integrity of RNA was assessed on an agarose gel. cDNA was synthesized from 1 μg of purified RNA, using 50 nM of Oligo dT, 16 mer, (Operon Biotechnologies SP230), 30 units of AMV Reverse Transcriptase (Promega M5108) and RNA-se free H_2_0 in a reaction volume of 25 μl, according to the manufacturer's recommendations. Identical reactions devoid of reverse transcriptase (-RT) were carried out in parallel and did not lead to any DNA amplification of predicted molecular weight in contrast to reverse transcriptase-containing reactions. Reactions containing H_2_0 instead of cDNA were also used in negative controls (data not shown).

A RT-PCR approach was used for the analysis of defensin expression in A549 and 16HBE human respiratory cell lines, as well as in primary culture of human respiratory cells exposed to RC, SC, or HF. Gene-specific primers for hBD1 and hBD2 were designed according to the sequences available at the National Center for Biotechnology Information http://www.ncbi.nlm.nih.gov/ in order to amplify specific cDNA sequences and avoid genomic DNA amplification. In this respect, primer sequences were designed to cover at least two subsequent exons, the human beta-defensin (HBD) -1 and -2 (NCBI accession # NM 005218.3 and NM 004942.2, respectively). It should be observed that hBD2 is now referred to as hBD-4 in the NCBI database. However, we decided to use the term, hBD2, since it is widely used in scientific literature today [53]. For the analysis of hBD8, hBD9 and hBD18, we relied on previous studies; the primers and PCR conditions were used as described in [10].

The PCR-amplified cDNA band of glyceraldehyde-3-phosphate dehydrogenase (GAPDH, NCBI accession # NM 2046.3) was used as an internal control with the predicted size of 473 bp. In each reaction, the initial denaturing step was 94°C for 8 min, followed by 32–38 cycles [denaturation at 94°C for 40 seconds, annealing at 56–61°C (according to primer melting temperature) for 40 s and elongation at 72°C for 1 minute]. The final elongation step was 72°C for 7 min. The primer annealing temperatures, cycles and predicted PCR product sizes for the transcripts investigated are summarised in Table 1. The PCR-amplified cDNA products were separated by electrophoresis on a 2% agarose gel and visualised by ethidium bromide after staining. The forward primers (f) and reverse primers (r) used are presented in Table 1. Identification of each defensin was confirmed by direct sequencing of respective PCR products, using upstream PCR primers (DNA Sequencing Facility, Qiagen, France).

### Quantitative Real Time PCR

The level of mRNA for HBD2, HBD9 and GAPDH in human cells was quantified using real time PCR analysis. Three different experiments were performed. Isolation of total RNA with TRIzol Reagent and synthesis of cDNA was performed as described above. To perform real time PCR, gene-specific primers were designed according to the sequences available at the National Center for Biotechnology Information http://www.ncbi.nlm.nih.gov/, using Beacon Designer 2 software (Table 2).

**Table 2:** Primer sequences and annealing temperatures (Real Time PCR)

Primers	Sequences	Conditions
hBD2f hBD2r	5'-tatctcctcttctcgttcctcttc-3' 5'-ccacaggtgccaatttgtttatac-3'	40 cycles, 55°C, 2.5% DMSO
hBD9f hBD9r	5'-ggcctaaatccaggtgtgaa-3' 5'-tcaaatgttggcaagtggag-3'	40 cycles, 55°C
GAPDHf GAPDHr	5'-acccactcctccacctttgac-3' 5'-tccaccaccctgttgctgtag-3'	40 cycles, 55°C

In order to amplify specific cDNA sequences and to avoid genomic DNA amplification, all primer sequences were designed to cover at least two subsequent exons (Table 2). Relative quantification relates the PCR signal of the target transcript in a treatment group to that of an untreated control. For each primer-pair, the amplification efficiency was determined by serial dilution experiments and the resulting efficiency coefficient was used for quantification of the products [54]. Each 25 μl Quantitative PCR mixture included 5 microl of DNA, 0.08 μl of primers (300 nM), 12.5 μl of CYBR green IQ supermix (2×) (ABgene) and H2O. Quantitative PCR amplification was carried out on an iCycler iQ system (Bio-Rad, Marne la Coquette, France) with the following parameters: 15 min at 95°C and 40 cycles of two steps consisting of 30s at 95°C, 30 s at 55°C. The relative quantification of the mRNA levels of the target genes was determined using the deltaCT – method [55]. Briefly, the amount of target was normalised to the endogenous reference gene GAPDH: deltaCT = CT (target gene) – CT (GAPDH) where CT represents the cycle number required to reach a defined threshold target abundance. The relative mRNA level was calculated as × deltaCT (x = Primer efficiency) (Pfaffl, 2001). All reactions were performed in triplicate and included a negative (-RT) control without reverse transcriptase.

### Neutralising anti-IL-1β antibody

Experiments designed to analyse the role of IL-1 β in *A. fumigatus*-induced defensin expression were performed using real time PCR. 5 × 10^6 ^of A549 or 16HBE cells were placed in six well plates in 1.5 ml of the corresponding medium and grown until confluence. The cells were divided into three groups. The cells of the first group were exposed to either *A. fumigatus *morphotypes or beads for 18 hours as described above. Neutralising anti-IL-1β antibody (10 μg/ml) was added to the cells of the second group prior exposure to *A. fumigatus *organisms or beads for the same period. The amount of neutralising antibody was equal to that used in the experiments devoted to the study of the role of Il-1β synthesized by the monocytes infected with Streptococci [56]. Normal mouse immunoglobulin (10 μg/ml) was used instead of neutralising antibody for the third group of cells. After collection of cells, RNA were isolated using TRIzol reagent and real time PCR was performed as described above.

### Immunofluorescence

Either A549 or 16HBE cells were seeded at 5 × 10^5 ^cells per well in 1 ml of DMEM/F12 on 18-mm-diameter cover slips (Marienfeld, Germany) in 12 well plates (Nunc, NuclonTM Surface) in triplicate and grown for 16 h at 37°C. After washing the cover slips with 5% BSA/PBS (BSA, Fraction V, Sigma), the cells were exposed to either 10^6 ^fixed conidia or to 20 μl of the fixed HF solution (20 mg of dry weight/ml), or 5 × 10^6 ^latex beads for 24 hours. The untreated cell culture was used as a negative control. The treatment with 20 ng of Il-1β, a well-known inductor of defensins [57], was used as a positive control. In some experiments, the cells were treated with 10 ng/ml of TNF-α. The cells were then fixed with freshly prepared 4% solution of paraformaldehyde for 30 min at 37°C, followed by permeabilisation in 0.05% of Triton/PBS solution. The slides were then incubated in 5% BSA/PBS, and then in a solution of 10% normal goat serum (Sigma). After washing, rabbit anti-human hBD2 (Peptide Institute 234) at a dilution of 1:250 was applied as a primary antibody overnight at 4°C, followed by incubation with FITC-labelled goat anti-rabbit secondary antibody (Sigma, Ac35-FITC) at a dilution of 1:300 for 4 hours at room temperature [58]. After washing, the cover slips were mounted on slides with ProLong antifade Vectashield (Vectashield, Biovalley). Samples were viewed with a Zeiss fluorescence microscope using ×400 magnification. For each sample, cells from five random fields were counted and the percentage of the cells stained with anti-defensin-2 antibody was calculated as the number of stained cells divided by the total number counted, multiplied by 100. The epithelial cells incubated with normal rabbit serum at a dilution of 1:250 (Sigma, 10 mg/ml) as a negative control showed no reactivity.

### Analysis of co-localisation of intracellular hBD-2 and *A. fumigatus *conidia or hyphal fragments

Co-localisation experiments were performed according to the method described by Botterel at al. with modifications [32]. After exposing the cells to 10^6 ^per millilitre of medium of RC, SC or 20 μl of the standard HF solution (35 mg of dry weight/ml) for 18 hours, the cells were fixed and permeabilised as indicated above. The cells were then labelled with primary rabbit anti-hBD2 antibody (Peptide Institute 234) at a dilution of 1:250 overnight at 4°C, followed by incubation with Tex Red-labelled goat anti-rabbit secondary antibody (Sigma) at a dilution of 1:300 for 1 hour at 37°C. After washing in PBS, the cover slips were mounted on slides with ProLong antifade Vectashield (Vectashield, Biovalley, USA). Samples were viewed with a Zeiss fluorescence microscope using ×400 magnification and the images were compared to the phase-contrast images in order to identify stained internalised *A. fumigatus *organisms.

### Detection of hBD2 in cell supernatants

Analysis of the hBD2 in cell supernatants was performed by sandwich-ELISA. Either A549 or 16HBE cells were seeded at 10^6 ^cells per well in 1 ml of DMEM/F12 in 12 well plates in triplicate and grown for 24 h at 37°C. Primary culture HNT cells were grown for 48 hours in BEGM medium as described above. The cells were then exposed to 10^6 ^per millilitre of medium of RC, SC or 20 μl of the standard HF solution (35 mg of dry weight/ml) for 18 hours. Cell supernatants were then centrifuged at 9000 g for 10 min at 4°C and analysed for the presence of hBD2 with a commercial ELISA kit (Antigenix America, Inc., NY, USA) according to the manufacturer's instructions. Briefly, a 96-well ELISA plate (Nunc, NY, USA) was coated with 100 μl of 0.5 μg/ml of capture anti-hBD2 antibody. The plate was sealed and incubated overnight at room temperature. After washing with phosphate buffer solution (PBS) containing 0.05% Tween 20, non-specific binding sites of the wells were blocked with 200 μl of 0.1% Bovine Serum Albumin (BSA)/PBS solution for 1 hour at room temperature. The wells were then washed again and 100 μl of cell supernatants or standard recombinant hBD2 in duplicate were added to the wells for 2 hours at room temperature. Serial dilutions of standard hBD2 from 10 ng/ml to 0.01 ng/ml were performed in diluent containing 0.1 BSA in 0.05% Tween 20/PBS. After washing, 100 μl of tracer biotinilated antibody was added to the wells at a concentration of 0.25 μg/ml for 2 hours at room temperature. The wells were then washed again and streptavidin-horse radish peroxidise solution at a concentration of 1 μg/ml was added for 30 minutes at room temperature, followed by intensive washing. Liquid chromogenic substrate (3, 3', 5, 5'-Tetramethyl-Benzidine) solution was used for colour development. The ELISA plate was read using a 96-well plate reader IEMS, THERMO LABSYSTEMS (Thermo Fisher Scientific, Cergy-Pontoise, France) at 450 nm (correction set at 650 nm) with 5 min intervals for 20 minutes. The sensitivity of ELISA for hBD2 was 10 pg/ml.

### Analysis of defensin expression by cells treated with inhibitors of protein synthesis and gene transcription

To examine the mechanism(s) for inducible defensin expression in response to *A. fumigatus*, human airway epithelial cells A549 or 16HBE were pre-treated with either 2.5 μg of cycloheximide (an inhibitor of protein synthesis) per ml, 0.5 μg of actinomycin D (an inhibitor of RNA transcription) per ml, or DMSO (vehicle control), 1 h before exposure to *A. fumigatus *for an additional 6 or 18 hours. In this study, we used lower doses of actinomycin D and cycloheximide than were previously described [33], in order to avoid their toxic effect during incubation of the cells for 18 hours. The viability of human cells as assessed by trypan blue and total RNA yield were checked after each treatment, and no differences were found between experimental and untreated control cells.

### Statistical analysis

The differences in the percentage of the cells positively stained with anti-defensin antibody in the cell cultures exposed or not to *A. fumigatus *were assessed by analysis of variance. P-values <0.05 were considered to be significant. Tukey's honestly significant difference test was applied for comparison of means between groups. The values are expressed as mean ± SEM. At least three different assays were performed per experiment

## Authors' contributions

LA, DH, NB and IM carried out PCR experiments, FF was responsible for cell growth, and FF and NB performed immunofluorescence experiments. DH was in charge of the preparation of *A. fumigatus *organisms. FF, MA and AC performed the experiments with live *A. fumigatus*. VTS and ABS were involved in primary culture cell growth. DG designed some of the primers, RC participated in the preparation of *A. fumigatus *mycelium and DH and NB carried out ELISA experiments. JPL participated in the design of some of the experiments. NB was responsible for the conception and design of the study, analysis and interpretation of the data, statistical analysis and for the writing of the manuscript. JPL and NB were responsible for revising the manuscript for intellectual content and gave the final approval of the version to be submitted. All authors read and approved the final version of the manuscript.

## References

[B1] Denning DW Anderson MJ Turner G Latgé JP Bennett JW Sequencing the *Aspergillus fumigatus *genome Lancet Infect Dis 2002 2 4 251 253 10.1016/S1473-3099(02)00243-8 11937425

[B2] Kleinberg M Aspergillosis in the CLEAR outcomes trial: working toward a real-world clinical perspective Med Mycol 2005 43 Suppl 1 289 294 10.1080/13693780400025237 16110822

[B3] Bals R Epithelial antimicrobial peptides in host defense against infection Respir Res 2000 1 141 150 11667978 10.1186/rr25 PMC59560

[B4] Ganz T Weiss T Antimicrobial peptides of phagocytes and epithelia Semin Hematol 1997 34 4 343 354 9347585

[B5] Cunliffe RN Mahida YR Expression and regulation of antimicrobial peptides in the gastrointestinal tract J Leukocyte Biol 2004 75 49 58 10.1189/jlb.0503249 14525966

[B6] Tang Y-Q Yaun J Osapay G Osapay C Tran D Miller C Quellette A Selsted M A cyclic antimicrobial peptide produced in primateleukocytes by the ligation of two truncated a-defensins Science 1999 286 498 502 10.1126/science.286.5439.498 10521339

[B7] García JR Jaumann F Schulz S Krause A Rodríguez-Jiménez J Forssmann U Adermann K Klüver E Vogelmeier C Becker D Hedrich R Forssmann WG Bals Identification of a novel, multifunctional beta-defensin (human beta-defensin 3) with specific antimicrobial activity. Its interaction with plasma membranes of Xenopus oocytes and the induction of macrophage chemoattraction Cell Tissue Res 2001 306 2 257 264 10.1007/s004410100433 11702237

[B8] Jia HP Schutte BC Schudy A Linzmeier R Guthmiller JM Johnson GK Tack BF Mitros JP Rosenthal A Ganz T McCray PB Discovery of new human beta-defensins using a genomics-based approach Gene 2001 263 1–2 211 218 11223260 10.1016/s0378-1119(00)00569-2

[B9] Schutte BC Mitros JP Bartlett JA Walters JD Jia HP Welsh MJ Casavant TLPB Jr McCray TL Discovery of five conserved beta-defensin gene clusters using a computational search strategy Proc Natl Acad Sci USA 2002 99 4 2129 33 11854508 10.1073/pnas.042692699 PMC122330

[B10] Kao CY Chen Y Zhao YH Wu R ORFeome-based search of airway epithelial cell-specific novel human [beta]-defensin genes Am J Respir Cell Mol Biol 2003 29 1 71 80 10.1165/rcmb.2002-0205OC 12600824

[B11] Bensch KW Raida M Mägert HJ Schulz-Knappe P Forssmann WG hBD-1: a novel beta-defensin from human plasma FEBS Lett 1995 368 2 331 335 10.1016/0014-5793(95)00687-5 7628632

[B12] Zhao C Wang I Lehrer RI Widespread expression of beta-defensin hBD-1 in human secretory glands and epithelial cells FEBS Lett 2005 396 2-3 319 322 8915011 10.1016/0014-5793(96)01123-4

[B13] Harder J Bartels J Christophers E Schröder JM A peptide antibiotic from human skin 1997 387 861 867 9202117 10.1038/43088

[B14] Schröder JM Harder J Human beta-defensin-2 Int J Biochem Cell Biol 1999 31 6 645 651 10.1016/S1357-2725(99)00013-8 10404637

[B15] Weinberg A Krisanaprakornkit S Dale BA Epithelial antimicrobial peptides: review and significance for oral applications Crit Rev Oral Biol Med 1998 9 4 399 414 **dendritic and T cell CCR6**. *Science *1999, **286**:525–528 10.1177/10454411980090040201 9825219

[B16] Funderburg N Lederman MM Feng Z Drage MG Jadlowsky J Harding CV Weinberg A Sieg SF Human -defensin-3 activates professional antigen-presenting cells via Toll-like receptors 1 and 2 Proc Natl Acad Sci USA 2007 104 47 18631 18635 18006661 10.1073/pnas.0702130104 PMC2141828

[B17] Soruri A Grigat J Forssmann U Riggert J Zwirner J Beta-Defensins chemoattract macrophages and mast cells but not lymphocytes and dendritic cells: CCR6 is not involved Eur J Immunol 2007 37 9 2474 2486 10.1002/eji.200737292 17705135

[B18] Yang D Chertov O Bykovskaia S Chen Q Buffo MJ Shogan J Anderson M Schröder JM Wang JM Howard OM Oppenheim JJ Beta-defensins: linking innate and adaptive immunity through dendritic and T cell CCR6 Science 286 525 528 10.1126/science.286.5439.525 10521347

[B19] Levitz SM Selsted ME Ganz T Lehrer RI Diamond RD In vitro killing of spores and hyphae of *Aspergillus fumigatus *and *Rhizopus oryzae *by rabbit neutrophil cationic peptides and bronchoalveolar macrophages J Infect Dis 1986 154 3 483 489 3525696 10.1093/infdis/154.3.483

[B20] Okamoto T Toyohiro T Wei B Ueta E Yamamoto T Osaki T Regulation of Fungal Infection by a Combination of Amphotericin B and Peptide 2, a Lactoferrin Peptide That Activates Neutrophils Clin Diagn Lab Immunol 2004 11 6 1111 1119 15539515 10.1128/CDLI.11.6.1111-1119.2004 PMC524744

[B21] Simon A Kullberg BJ Tripet B Boerman OC Zeeuwen P Ven-Jongekrijg J van der Verweij P Schalkwijk J Hodges R Meer JW van der Netea MG Drosomycin-like defensin, a human homologue of Drosophila melanogaster drosomycin with antifungal activity Antimicrob Agents Chemother 2008 52 4 1407 1412 18212107 10.1128/AAC.00155-07 PMC2292511

[B22] Berkova N Lair-Fulleringer S Femenia F Huet D Wagner MC Gorna K Tournier F Ibrahim-Granet O Guillot J Chermette R Boireau P Latge JP Aspergillus fumigatus conidia inhibit tumour necrosis factor- or staurosporine-induced apoptosis in epithelial cells Intern Immunol 2006 18 139 150 10.1093/intimm/dxh356 16357007

[B23] Khoufache K Puel O Loiseau N Delaforge M Rivollet D Coste A Cordonnier C Escudier E Botterel F Bretagne S Verruculogen associated with *Aspergillus fumigatus *hyphae and conidia modifies the electrophysiological properties of human nasal epithelial cells BMC Microbiol 2007 23:7 5 16 10.1186/1471-2180-7-5 PMC1797047 17244350

[B24] Zhang Z Liu R Noordhoek JA Kauffman HF Interaction of airway epithelial cells (A549) with spores and mycelium of *Aspergillus fumigatus* J Infect 2005 51 5 375 82 10.1016/j.jinf.2004.12.012 16321648

[B25] Bellocchio S Bozza S Montagnoli C Perruccio K Gaziano R Pitzurra L Romani L Immunity to Aspergillus fumigatus: the basis for immunotherapy and vaccination Med Mycol 2005 43 S181 188 10.1080/14789940500051417 16110810

[B26] Steele C Rapaka RR Metz A Pop SM Williams DL Gordon S Kolls JK Brown GD The beta-glucan receptor dectin-1 recognizes specific morphologies of Aspergillus fumigatus PLoS Pathog 2005 1 4 42 48 10.1371/journal.ppat.0010042 PMC1311140 16344862

[B27] Mambula SS Sau K Henneke P Golenbock DT Levitz SM Toll-like receptor (TLR) signaling in response to *Aspergillus fumigatus* J Biol Chem 2002 277 42 39320 39326 10.1074/jbc.M201683200 12171914

[B28] Stark H Roponen M Purokivi M Randell J Tukiainen H Hirvonen MR *Aspergillus fumigatus *challenge increases cytokine levels in nasal lavage fluid Inhal Toxicol 2006 18 13 1033 1039 10.1080/08958370600904579 16966303

[B29] Wang JE Warris A Ellingsen EA Jorgensen PF Flo TH Espevik T Solberg R Verweij PE Aasen AO Involvement of CD14 and Toll-Like Receptors in Activation of Human Monocytes by *Aspergillus fumigatus *Hyphae Infect Immun 2001 69 4 2402 2406 11254600 10.1128/IAI.69.4.2402-2406.2001 PMC98172

[B30] Wasylnka J Moore M Uptake of *Aspergillus fumigatus *Conidia by Phagocytic and Nonphagocytic Cells In Vitro: Quantitation Using Strains Expressing Green Fluorescent Protein Infect Immun 2002 70 3156 3163 12011010 10.1128/IAI.70.6.3156-3163.2002 PMC127978

[B31] Wasylnka JA Moore MM Aspergillus fumigatus conidia survive and germinate in acidic organelles of A549 epithelial cells J Cell Sci 2003 116 8 1579 1587 10.1242/jcs.00329 12640041

[B32] Botterel F Gross K Ibrahim-Granet O Khoufache K Escabasse V Coste A Cordonnier C Escudier E Bretagne S Phagocytosis of *Aspergillus fumigatus *conidia by primary nasal epithelial cells in vitro BMC Microbiol 2008 18;8 97 106 10.1186/1471-2180-8-97 PMC2440385 18564423

[B33] Krisanaprakornkit S Kimball JR Weinberg A Darveau RP Bainbridge BW Dale BA Inducible expression of human beta-defensin 2 by *Fusobacterium nucleatum *in oral epithelial cells: multiple signaling pathways and role of commensal bacteria in innate immunity and the epithelial barrier Infect Immun 2000 68 5 2907 2915 10768988 10.1128/IAI.68.5.2907-2915.2000 PMC97503

[B34] Singh PK Jia HP Wiles K Hesselberth J Liu L Conway BA Greendberg EP Valore EV Welsh MJ Ganz T Tack BF McGray PB Jr Production of beta-defensins by human airway epithelia Proc Natl Acad Sci USA 1998 95 25 14961 14966 9843998 10.1073/pnas.95.25.14961 PMC24558

[B35] Rivas-Santiago B Schwander SK Sarabia K Diamond G Klein-Patel ME Hernandez-Pando R Ellner JJ Sada E Human β-defensin 2 is expressed and associated with *Mycobacterium tuberculosis *during infection of human alveolar epithelial cells Infect Immun 2005 73 4505 4511 16040961 10.1128/IAI.73.8.4505-4511.2005 PMC1201238

[B36] Premratanachai P Joly S Johnson GK McCray PB Jia HP Guthmiller JM Expression and regulation of novel human beta-defensins in gingival keratinocytes Oral Microbiol Immunol 2004 19 2 111 117 10.1111/j.0902-0055.2002.00127.x 14871351

[B37] Bhat S Song YH Lawyer C Milner S Modulation of the Complement System by Human β-Defensin 2 J Burns Wounds 2007 5 e10 17235375 PMC1769518

[B38] Perlmutter DH Colten HR Crystal RG, West JB The role of complement in the pathophysiology of lung diseases The lung 1997 2 Philadelphia: Lippincott-Raven 841 57

[B39] Varsano S Kaminsky M Kaiser M Rashkovsky L Generation of complement C3 and expression of cell membrane complement inhibitory proteins by human bronchial epithelium cell line Thorax 2000 55 364 369 10770816 10.1136/thorax.55.5.364 PMC1745745

[B40] Gersuk GM Underhill DM Zhu L Marr KA Dectin-1 and TL Rs permit macrophages to distinguish between different *Aspergillus fumigatus *cellular states J Immunol 2006 176 6 3717 3724 16517740 10.4049/jimmunol.176.6.3717

[B41] Daher KA Lehrer RI Ganz T Kronenberg M Isolation and characterization of human defensin cDNA clones Proc Natl Acad Sci USA 1988 85 7327 7332 3174637 10.1073/pnas.85.19.7327 PMC282179

[B42] Rahman A Fahlgren A Sitohy B Baranov V Zirakzadeh A Hammarström S Danielsson A Hammarström ML Beta-defensin production by human colonic plasma cells: a new look at plasma cells in ulcerative colitis Inflamm Bowel Dis 2007 13 7 847 855 10.1002/ibd.20141 17387677

[B43] Rizzo A Paolillo R Buommino E Lanza AG Guida L Annunziata M Carratelli CR Modulation of cytokine and beta-defensin 2 expressions in human gingival fibroblasts *infected with *Chlamydia pneumoniae Int Immunopharmacol 2008 8 9 1239 1247 10.1016/j.intimp.2008.04.015 18602070

[B44] Lopes Bezerra L Filler S Interactions of *Aspergillus fumigatus *with endothelial cells:internalization, injury, and stimulation of tissue factor activity Blood 2004 103 2143 2149 10.1182/blood-2003-06-2186 14630829

[B45] Mehrad B Strieter RM Standiford TJ Role of TNF-alpha in pulmonary host defense in murine invasive aspergillosis J Immunol 1999 162 1633 40 9973423

[B46] Netea MG Warris A Meer JW Van der Fenton MJ Verver-Janssen TJ Jacobs LE Andresen T Verweij PE Kullberg BJ Aspergillus fumigatus evades immune recognition during germination through loss of toll-like receptor-4-mediated signal transduction J Infect Dis 2003 188 320 6 10.1086/376456 12854089

[B47] Behnsen J Hartmann A Schmaler J Gehrke A Brakhage A Zipfel PF The opportunistic human pathogenic fungus *Aspergillus fumigatus *evades the host complement system Infect Immun 2008 76 2 820 827 18039838 10.1128/IAI.01037-07 PMC2223477

[B48] Lieber M Smith B Szakal A Nelson-Rees W Todaro S A continuous tumor-cell line from a human lung carcinoma with properties of type II alveolar epithelial cells Int J Cancer 1976 17 62 67 10.1002/ijc.2910170110 175022

[B49] Cozens AL Yezzi MJ Kunzelmann K Ohrui T Chin L Eng K Finkbeiner WE Widdicombe JH Gruenert DC CFTR expression and chloride secretion in polarized immortal human bronchial epithelial cells Am J Respir Cell Mol Biol 1994 10 1 38 47 7507342 10.1165/ajrcmb.10.1.7507342

[B50] Million K Tournier F Houcine O Ancian P Reichert U Marano F Effects of retinoic acid receptor-selective agonists on human nasal epithelial cell differentiation Am J Respir Cell Mol Biol 2002 25 6 744 750 10.1165/ajrcmb.25.6.4549 11726401

[B51] Morigi M Zoja C Colleoni S Angioletti S Imberti B Donadelli R Remizzi A Xenogeneic Serum Promotes Leukocyte-Endothelium Interaction under Flow through Two Temporally Distinct Pathways: role of complement and nuclear factor-kappaB J Am Soc Nephrol 1999 10 2197 2203 10505697 10.1681/ASN.V10102197

[B52] Griese M Reinhardt D Smaller sized particles are preferentially taken up by alveolar type II pneumocytes J Drug Target 1998 5 471 479 9783678 10.3109/10611869808997873

[B53] Krisanaprakornkit S Chotjumlong P Kongtawelert P Reutrakul V Involvement of phospholipase D in regulating expression of anti-microbial peptide human beta-defensin-2 Int Immunol 2008 20 1 21 29 10.1093/intimm/dxm115 17986621

[B54] Pfaffl MW A new mathematical model for relative quantification in real-time RT-PCR Nucleic Acids Res 2001 29 9 e45 11328886 10.1093/nar/29.9.e45 PMC55695

[B55] Livak KJ Schmittgen TD Analysis of relative gene expression data using real-time quantitative PCR and the 2(-Delta Delta C(T)) Methods 2001 25 402 408 10.1006/meth.2001.1262 11846609

[B56] Hahn CL Best AM Tew JG Rapid tissue factor induction by oral streptococci and monocyte-IL-1beta J Dent Res 2007 86 3 255 259 10.1177/154405910708600311 17314258

[B57] Jang BC Lim KJ Choi IH Suh MH Park JG Mun KC Bae JH Shin DH Suh SI Triptolide suppresses interleukin-1beta-induced human beta-defensin-2 mRNA expression through inhibition of transcriptional activation of NF-kappaB in A549 cells Int J Mol Med 2007 19 5 757 763 17390080

[B58] Sugawara Y Uehara A Fujimoto Y Kusumoto S Fukase K Shibata K Sugawara S Sasano T Takada H Toll-like Receptors, NOD1, and NOD2 in Oral Epithelial Cells J Dent Res 2006 85 6 524 529 10.1177/154405910608500609 16723649

